# Social support from family, associated factors and relationship with glycemic control among diabetic patients in Uganda: a cross-sectional study

**DOI:** 10.11604/pamj.2023.45.72.38256

**Published:** 2023-06-01

**Authors:** Jude Tadeo Onyango, Jane Frances Namatovu, Innocent Kabahena Besigye, Mark Kaddumukasa, Scovia Nalugo Mbalinda

**Affiliations:** 1 Department of Family Medicine, Makerere University, P.O. Box 7062, Kampala, Uganda,; 2 Department of Medicine, Makerere University, P.O. Box 7062, Kampala, Uganda,; 3 Department of Nursing, Makerere University, P.O. Box 7062, Kampala, Uganda

**Keywords:** Social support, family, diabetes, glycemic control, Uganda

## Abstract

**Introduction:**

social support from family members in diabetes management is a predictor of optimal glucose control. There is limited evidence of the relationship in Uganda. The objective was to determine association of social support from family and glycemic control, and association of social demographic and clinical characteristics with family support among diabetic patients in eastern Uganda.

**Methods:**

this was a cross-sectional study involving 405 adult patients attending diabetic clinics between May 2021 and June 2021. Socio-demographics, clinical characteristics, social support from family, and glycemic control data were collected. Descriptive statistics were done and associations were determined using Pearson chi-square and Fisher´s exact tests. Generalized linear model was used to determine independent association with social support from family.

**Results:**

the mean age was 52 years, (60%) were female, majority (49.4%) were 45-64 years old. Perceived social support from family (PSS-fa) and good glucose control were found in; (95.3%) and (20.99%) respectively. PSS-fa was associated with good glucose control. Financial contribution from family members to cost of care, cohesion among family members in support of care, being (married/cohabiting) and monthly income ≥28 USD were associated with PSS-fa. Factors independently associated with PSS-fa were; female gender, financial contribution to cost of care and cohesion among family in support of care.

**Conclusion:**

social support from family was associated with good glycemic control. Factors associated with PSS-fa were; female gender, financial contribution from family to cost of care and cohesion among family in support of care.

## Introduction

Optimal management of Diabetes Mellitus (DM) has daily requirements on the individual that are not optional; adherence to medication, diet and exercise recommendations, self-testing, and follow up. This can be challenging to the sufferer resulting in emotional, mental and psychological impact [1]. The family setting creates the practical, social and emotional context for self-care where these requirements can be met and is where majority of diabetes care takes place. The people in the immediate social context motivate and actively support patient self-management on an ongoing basis. The result is an increased likelihood of optimal glycemic control and prevention or delay of incidence of complications responsible for morbidity and mortality associated with the disease [2]. The evidence from studies around the world has shown a positive influence of diabetes specific supportive behaviour and actions from family members on individuals´ self-management behavior and glucose control [3-8]. Also, negative effects on glycemic control have been documented with non-supportive behavior and actions, and non-cohesive and dysfunctional family environments [5,9-11]. The patient´s perception of whether support is provided or is available has been used as an indirect measure of social support from family in a number of studies conducted around the world including Africa [12-15]. This study intends to extend the existing evidence to Uganda´s setting by determining the association between perceived social support from family and glycemic control.

## Methods

**Study design:** the design was cross-sectional and used quantitative methods.

**Study period:** three months from May 2021 to July 2021 inclusive.

**Study setting:** diabetes outpatient clinics of three hospitals in Uganda´s eastern region. Each of the hospitals acts as the referral health facility for lower-level health facilities in their catchment area. Each hospital had a weekly clinic day when diabetes patients are reviewed.

**Study population:** consisted of participants who were diabetic, of age 18 years and above who had been registered with the diabetic clinic for at least six months. They should have been living with other people they consider as family members and should be able to speak any of six commonly spoken languages in the eastern region of Uganda. The study excluded those who were pregnant, too weak to participate in the interview, and those who had major psychiatric conditions with the possibility of impaired perception of family support.

**Estimated sample size:** a sample size of 405 participants was used. This was determined using 5% as the level of significance, power of 80% [16]. The proportion of diabetic patients with family support who achieve good glycemic control was anticipated at 60%. The proportion of diabetic patients with no family support who achieve good glycemic control was anticipated at 45%. Ten percent (10%) was the estimate for possible incomplete information.

**Sampling procedure:** probability proportionate to size sampling was applied. The three hospitals´ medical records show the number of patients registered with the diabetic clinics as; 1216, 1515, and 610 for Soroti, Mbale and Jinja respectively. Each of the hospitals contributed a sample size proportion determined by the ratio of average patient attendance per clinic day; (120 Jinja RRH: 150 Mbale RRH: 150 Soroti RRH). The total sample size of 405 was made up of; Jinja RRH - 105 patients, Mbale RRH-150 patients, and Soroti RRH - 150 patients. Using systematic random sampling, participants were selected to make up the sample size fraction at each clinic. Sampling intervals of twelve and six were used at (Mbale and Soroti) RRHs and Jinja RRH hospital respectively.

**Data collection and study variables:** a structured questionnaire developed through review of the literature was used to collect data. The first part of the questionnaire was for social demographic and clinical characteristics and had thirty-five items. The second part consisted of items adopted from the original perceived social support from family questionnaire (PSS-fa) that together with the scale [12]. The PSS-fa scale consists of twenty items that assess perceived social support from family members. The PSS-Fa has been found to have good reliability and validity. It had been used before for studies in other African populations to assess social support [15,17,18]. In its development and validation process, the scale showed very good psychometric properties with a Cronbach´s alpha coefficient of 0.90 indicating excellent internal consistency. A prior pre-test of the questionnaire containing the 2 parts, was done in each of the three hospitals among twenty diabetic patients (with similar inclusion criteria) not involved in the final sample to ensure clarity of the questions. The pre-tested questionnaire was then administered by three priorly trained research assistants to the study participants in the waiting area of the clinic before their medical consultation. The research assistants were nurses employed in the hospital but not involved with diabetic clinic. In addition, the nurses were holders of a diploma certificate in nursing (Registered level nurses) and proficient in English and at least one of five languages spoken in the region (Lumasaba, Lusoga, Jopadhola, Ateso and Kiswahili) served by the three hospitals. The interview would take about twenty to 30 minutes and thereafter the study participant was helped to access clinical care.

**Data entry and analysis software:** versions of EpiData 3.1 and STATA 15 were used for data entry and data analysis respectively.

**Operational definitions:** perceived social support was categorized into three; “Strong”, “Weak”, and “No” perceived social support. “Strong” if PSS-fa scale score was above or equal to 11, “weak” if score was from (7 up to 10), and “No” if score was below or equal to 6. A second categorization was done to test the hypothesis of the study. Two categories of; “Strong” and “Weak” together for those with perceived social support (score 7 and above), and “No” perceived family support (6 and below). To estimate glycemic control, an average of the last three fasting blood glucose (FBG) levels measured monthly was determined for each participant. The average FBG was categorized into two; average FBG ≤ 7.2 mmol/L for “Good” glycemic control and average FBG > 7.2 mmol/L for “Poor” glycemic control.

**Data analysis:** the descriptive statistics (mean, median, frequencies, percentages) were applied for description of variables, were necessary. Pearson Chi and Fisher´s exact tests were applied to determine associations between (socio-demographic and clinical variables) and average FBG. Fisher´s exact test was applied to determine the association between perceived social support from family (PSS-fa) and average FBG. Independent association of socio-demographic and clinical variables with perceived social support was determined using generalized linear modeling. A P-value of 0.05 or less was considered significant.

**Ethical approval:** the study was approved by Ethics and Research committee of the School of Medicine (SOMREC), Makerere University College of Health Sciences (#REC REF 2020-142).

**Consent to participate:** informed written consent was obtained from all study participants. Study code numbers instead of participant names were used to identify questionnaires. Access to the study participants´ information was only possible to the principal investigator and study team.

## Results

Four hundred and five adult diabetic patients participated in the study. Their average age was 52 (SD14.9) years with females making up the majority (60%). The majority of the sample were; Christian (79.3%), married or cohabiting (67%), and had completed primary education (78.3%). Most originated (89.6%) or resided within the eastern region (99%). As far as employment was concerned, (81.2%) were employed. The median monthly income of the sample was 28 [8,56] USD. The participants´ families had a median number of people employed as 1 [0,2]. Over half (55.9%) were living in extended family settings, with a median house hold size of 7 family members. Almost all participants had domestic help in different forms related to managing diabetes. In regard to cohesiveness among family members in support of the affected individual, over half reported some or high cohesion. However, majority (64.2%) reported limited (minimal or no) financial contribution from family members to costs of hospital care.

Very few (7.7%) participants had Type I diabetes. The rest (92.3%) had Type II diabetes. The median duration with diabetes and drug treatment was 5 [2,10] and 4 [2,9] years respectively. Over half (56.0%) used insulin entirely or partly for treatment of their diabetes. Most participants could access a functional Glucometer (74.1%) outside the hospital and only (11.9%) owned one. In the case of diabetes education, almost all (95.5%) had received Diabetes Self-Management Education (DSME) and most (73.1%) had family members who had received DSME. However, only (21%) possessed or used documents as a reference for guiding decision making in the management of their diabetes at home. Regarding other chronic ailments, just over half (50.9%) reported having at least one other chronic illness. The majority (75.1%) adhered to the prescribed monthly clinic attendance. About (12%) had been admitted to hospital because of their diabetes at least once in the last 6 months. Among the socio demographic characteristics, there was a statistical association with good glycemic control for participants; of older age, of higher monthly income and from families with greater number of members gainfully employed. There was no difference observed on glycemic control for participants´; gender, marital status, or level of education completed. In addition, distance from their residence to hospital, house hold size, living arrangement at home, whether they received domestic help for their diabetes management or financial contribution for their hospital care costs from family members, and existence of cohesion among family members to provide support in managing their condition had no statistical association with glycemic control (Table 1).

**Table 1:** bivariate analysis of socio-demographic characteristics and mean fasting blood sugar levels

	Poor GC (>7.2 MMOL/L)	Good GC (<=7.2MMOL/L)	Overall	
	(N = 320)	(N = 85)	(N = 405)	p-value
**Gender**				0.618**
Male	130(80.2%)	32(19.8%)	162 (40.0%)	
Female	190(78.2%)	53 (21.8%)	243(60.0%)	
**Age (in complete Years)**				<0.001***
18-44	108(33.3%)	17(21.0%)	125(30.9%)	
45-64	167(51.5%)	37(45.7%)	204(50.4%)	
> 65	49(15.2%)	27(33.3%)	76(18.8%)	
**Marital status**				0.149**
Married/cohabiting	220 (81.5%)	50 (18.5%)	270 (67.0%)	
Separated/Divorced /Widowed/Widower	70 (72.2%)	27 (27.8%)	97 (24.1%)	
Single/Never married	29(80.6%)	7(19.4%)	36(8.9%)	
**Highest level of formal education (completed)**				0.217**
None	67 (76.1%)	21 (23.9%)	88 (21.7%)	
Primary	142 (84.0%)	27 (16.0%)	169 (41.7%)	
Secondary	77 (74.8%)	26 (25.2%)	103 (25.4%)	
Tertiary/University	34(75.6%)	11(24.4%)	45(11.1%)	
**Distance from residence to Hospital**				0.798***
**<5Km**	68(21.0%)	17(21.0%)	85(21%)	
**5-10Km**	108(33.3%)	30(37.0%)	138(34.1%)	
**>10Km**	148(148(45.7%)	34(42.0%)	182(44.9%)	
**Employment status**				0.425**
formally employed	19(73.1%)	7(26.9%)	26 (6.4%)	
informal/self-employed/peasant	244 (80.5%)	59 (19.5%)	303 (74.8%)	
Unemployed	57 (75.0%)	19 (25.0%)	76 (18.8%)	
**Income per month (USD)**				0.041***
< 28 USD	237(73.2%)	50(61.7%)	287(70.9%)	
≥ 28 USD	87(26.8%)	31(38.3%)	118(29.1%)	
**House hold size**				0.564***
< 5 people	60(18.5%)	17(21.0%)	77(19.0%)	
5-10 people	195(60.2%)	46(56.8%)	241(59.5%)	
>10 people	69(21.3%)	18(22.2%)	87(21.5%)	
**No. employed in the home**				0.009***
**< 1**	94(29.0%)	12(14.8%)	106(26.2%)	
**1-2**	169(52.2%)	47(58.0%)	216(53.3%)	
**≥ 3**	61(18.8%)	22(27.2%)	83(20.5%)	
**Living arrangement**				0.710**
Nuclear family only	83(80.6%)	20(19.4%)	103(25.9%)	
extended family	175(78.8%)	47(21.2%)	222 (55.9%)	
Polygamous family	32(84.2%)	6(15.8%)	38(9.6%)	
Single parent family	25(73.5%)	9 (26.5%)	34(8.6%)	
**Domestic help from family**				1.000*
Yes	9(81.8%)	2(18.2%)	11 (2.7%)	
No	311(78.9%)	83 (21.1%)	394 (97.3%)	
**Financial contribution from family to the cost of care**				0.401**
No/Non	45(83.3%)	9(16.7%)	54(13.3%)	
Minimal	157(76.2%)	49(23.8%)	206 (50.9%)	
Most	69(84.1%)	13(15.9%)	82(20.2%)	
All	49 (77.8%)	14 (22.2%)	63 (15.6%)	
**cohesion among family**				0.912*
No/Non	16(84.2%)	3(15.8%)	19 (4.7%)	
Minimal	119 (79.3%)	31(20.7%)	150(37.0%)	
some amount	62(80.5%)	15(19.5%)	77(19.0%)	
highly cohesive	123 (77.4%)	36 (22.6%)	159 (39.3%)	

** Pearson Chi-square test*** µ Prevalence ratio * Fisher’s exact Chi-test

Of the clinical characteristics, there was a statistical association with good glycemic control for; use of documents for guidance/reference when managing diabetes, having other family members who had ever received DSME, and having access to a functional glucometer. In addition, having no other chronic disease apart from diabetes was also associated with good glucose control. However, there was no statistical difference in glycemic control observed for; type of diabetes, the duration with disease and/or treatment, the participant ever having received DSME, frequency of hospital visits, and whether a participant had been hospitalized for diabetes in the last 3 months (Table 2). A big majority was observed for strong social support from family (88.6%). Only 85 (20.99%) participants had good glycemic control (mean FBG level <7.2MMOL/L). The study found a statistical association with mean fasting blood glucose levels (p value-0.036). The proportion of those with mean FBG levels (<7.2 MMOL/L) who reported family support was significantly different from those who had mean glucose levels (<7.2 MMOL/L) who reported family support in managing their diabetes condition (Table 3).

**Table 2:** bivariate analysis of clinical characteristics and fasting blood sugar levels

	Poor GC (>7.2 MMOL/L)	Good GC (<=7.2MMOL/L)	Overall	
	(N = 320)	(N = 85)	(N = 405)	p-value
**Type of diabetes (documented)**				0.358*
Type I DM	27 (87.1%)	4 (12.9%)	31 (7.7%)	
Type II DM	293(78.3%)	81 (21.7%)	374(92.3%)	
Duration with diabetes (Years)				
< 5 years	158(48.7%)	43(53.1%)	201(49.6%)	0.349***
5-10 years	102(31.5%)	21(25.9%)	123(30.4%)	
>10 years	64(19.8%)	17(21.0%)	81(20%)	
Duration on drugs/treatment				0.930***
< 5 years	161(49.6)	44(54.3)	205(50.6%)	
5-10 years	99(30.6)	20(24.7)	119(29.4%)	
>10 years	64(19.8)	17(21.0)	81(20.0%)	
**Access to a functional Glucometer:**				0.013**
Own one	34 (70.8%)	14 (29.2%)	48 (11.9%)	
Don't own but can access one	193(76.6%)	59(23.4%)	252(62.2%)	
Can't access one	93 (88.6%)	12 (11.4%)	105 (25.9%)	
**Ever received Diabetes Self-Management Education (DSME)**				1.000*
Yes	309 (79.0%)	82 (21.0%)	391 (96.5%)	
No	11 (78.6%)	3 (21.4%)	14 (3.5%)	
**Family member/s ever had diabetes self-management education**				0.012**
Yes	77 (70.6%)	32 (29.4%)	109 (26.9%)	
No	243 (82.1%)	53 (17.9%)	296 (73.1%)	
**Have/use documentation to guide or refer to in managing diabetes**				0.018**
Yes	60(69.8%)	26(30.2%)	86(21.2%)	
No	260 (81.5%)	59 (18.5%)	319 (78.8%)	
**Has other chronic diseases apart from diabetes**				<0.001**
Yes	148(71.8%)	58(28.2%)	206(50.9%)	
No	172 (86.4%)	27(13.6%)	199(49.1%)	
**Frequency of hospital visits in the last six months**				0.202**
<=Monthly	260 (80.2%)	64 (19.8%)	324 (80.2%)	
Beyond a month	59 (73.8%)	21(26.3%)	80(19.8%)	
**Hospitalized in the last 3 months for diabetes**				0.124**
No	278(77.9%)	79(22.1%)	357(88.1%)	
Yes	42 (87.5%)	6 (12.5%)	11.9%)	

** Pearson Chi-square test ***Prevalence ratio * Fisher’s exact Chi-test

**Table 3:** bivariate analysis of perceived social support from family and Fasting Blood Glucose (FBG) levels

	Poor GC (>7.2 MMOL/L)	Good GC (<=7.2MMOL/L)	Total	
	(N = 320)	(N = 85)		p-value
Perceived social support from family - PSS-Fa sub scale				0.036*
<=6, No support	18(5.6%)	1(1.2%)	19(4.7%)	
7-10, Weak	25(7.8%)	2(2.4%)	27(6.7%)	
>11, Strong	277(86.6%)	82(96.5%)	359(88.6%)	

* Fisher’s exact Chi-test

The bivariate analysis showed a statistically significant difference in proportions comparing those with no perceived social support from family and those with perceived social support from family (weak/strong) for the following variables; having an intimate partner, receiving financial contribution from family to cater for their diabetes care costs, cohesion among family members towards care needs of the patient, and the patient´s average monthly income. There was no difference in perception of social support from family for variables of age, gender, education level, employment status, employment status of other family members, household size, living arrangement with other family members, type and duration of diabetes, and whether the patient had other comorbidities (Table 4). However; on multivariable analysis, female gender was independently associated with perception of social support from family. In addition, only financial contribution from family to cost of diabetic care, and cohesion among family members remained independently associated with perception of social support from family after adjusting for possible confounders (Table 5).

**Table 4:** bivariate analysis of social demographic, clinical characteristics and perceived social support from family (N=405)

Parameter	No Supportï¿½ n (%)	Weak/Strong Support n (%)	PR (95% CI)	p-value
**Social demographic characteristics**				
**Age (completed years)**				
18-44	6(31.6)	134(34.7)	1.00	
45-64	11(57.9)	189(49.0)	0.99(0.94-1.04)	0.606
≥65	2(10.5)	63(16.3	1.01(0.96-1.07)	0.659
**Gender**				
Male	5(26.3)	157(40.7)	1.00	
Female	14(73.7)	229(59.3)	0.97(0.93-1.01)	0.187
**Education level completed**				
No education/Primary	13(68.4)	245(63.5)	1.00	
O level/ A level/Tertiary/University	6(31.6)	141(36.5)	1.01(0.97-1.06)	0.653
**Marital status**				
Married/Cohabiting	6(31.6)	264(68.4)	1.00	6(31.6)
Widowed/ Divorced/ Separated/ Single	13(68.4)	122(31.6)	0.92(0.87-0.98)	**0.008**
**Employment status**				
Employed	15(79.0)	314(81.4)	1.00	
Unemployed	4(29.0)	72(18.6)	0.99(0.94-1.05)	0.803
**House hold size**				
<5 people	7(36.8)	70(18.1)	1.00	
5-10 people	10(52.6)	231(59.8)	1.05(0.98-1.14)	0.169
>10 people	2(10.5)	85(22.1)	1.07(0.99-1.16)	0.069
**Other family members employed**				
No	9(47.4)	99(25.6)	1.00	
Yes	10(52.6)	287(74.4)	1.05(0.99-1.12)	0.089
**Estimate of average income per month**				
<28 USD	17(89.5)	270(70.0)	1.00	
>28 USD	2(10.5)	116(30.0)	1.04(1.05-1.08)	**0.022**
**Living arrangement with other family members**				
Nuclear	5(26.3)	97(25.2)	1.00	
Extended/Polygamous	8(42.1)	253(65.5)	1.02(0.97-1.07)	0.445
Single parent	6(31.6)	36(9.3)	0.90(0.79-1.03)	0.121
**Financial contribution from family to cost of care**				
Most/All	12(63.1)	43(11.1)	1.00	
Minimal	6(31.6)	199(51.6)	1.24(1.08-1.43)	**0.003**
None	1(5.3)	144(37.3)	1.27(1.10-1.46)	**0.001**
**Level of cohesion among family members**				
Some/High	9(47.4)	10(2.6)	1.00	
Minimal	8(42.1)	141(36.5)	1.80(1.17-2.76)	0.007
None	2(10.5)	235(60.9)	1.88(1.23-2.89)	**0.004**
**Clinical characteristics**				
**Type of Diabetes**				
Type I	15(79.0)	359(93.0)	1.00	
Type II	4(21.0)	27(7.0)	0.91(0.79-1.04)	0.165
**Duration with Diabetes**				
<5 years	10(52.6)	191(49.5)	1.00	
5-10 years	3(15.8)	120(31.1)	1.03(0.98-1.07)	0.222
>10 years	6(31.6)	75(19.4)	0.97(0.91-1.04)	0.464
**Has other chronic diseases**				
Yes	7(36.8)	199(51.6)	1.00	
No	12(63.2)	187(48.4)	0.97(0.93-1.02)	0.214

PR: Prevalence Ratio

**Table 5:** multivariable analysis of socio-demographic and clinical characteristics associated with perceived support from family (weak/strong)

Parameter	aPR	95% CI	p-value
**Gender**			
Male	1.00		
Female	0.96	0.92-0.99	**0.044**
**Financial contribution of family to cost of care**			
Most/All	1.00		
Minimal	0.99	0.95-1.03	0.651
None	0.90	0.82-1.00	**0.043**
**Level of cohesion among family members**			
Some cohesion/highly cohesive	1.00		
Minimal	0.96	0.92-1.01	0.128
None	0.58	0.38-0.88	**0.011**
**Type of Diabetes**			
Type II	1.00		
Type I	0.89	0.80-1.01	0.062

aPR: adjusted Prevalence Ratio

## Discussion

This is the first survey that has examined the relationship between social support from family members and glycemic control among patients living with diabetes in Uganda. The study was conducted among 405 patients attending the diabetes clinics of three referral hospitals in eastern region of Uganda. The results reveal that a majority of participants had social support from family with the biggest proportion having a strong perception of social support. The findings also show that nearly all participants received some form of domestic help and majority of them reported high cohesion among family members in support of the affected individual. This finding is expected in the setting where the study was done, considering the prevailing family and cultural system that places certain obligations on other family or community members to assist in whatever form possible when one of them falls sick. Uganda is not unique in this regard. The same finding has been obtained in other countries. In Nigeria [17] and Burkina Faso [19] which have comparable family and cultural settings as well as Iran [20], strong family ties with accompanying obligations to the members especially in times of distress are a common finding. It is therefore not surprising that the finding of family support in the three countries is consistent with the present study.

The present study however shows that financial contribution from family members was minimal despite the participants scoring highly on perceived social support. This could be explained by the generally low economic status (mean monthly of 50.1USD) of majority participants and their families, and the resulting limited ability to afford the necessities to facilitate optimal care considering the prevailing relatively high financial costs that were to be incurred in care for; food, transport to hospital, and all or part of their medication among other needs. The mean number of people in the home who had some form of employment was 1.5 (1.32) which means that there were not many other people in the family setting who could make a financial contribution to support care activities because they were of a similar or less economic standing. The situation is made worse when there are financial needs from other people in the family to be catered for as shown by the relatively large house hold size (mean of 8.1 SD4.51), majority living in an extended or polygamous family arrangement. Indeed, there is evidence that low income affects provision of effective family support and limits implementation of self-management activities resulting in poor glycemic control [21-25]. In this study, only (20.99%) of study participants had good glucose control. A similar finding has also been observed in one study conducted in the western part of Uganda [26] were only (15.7%) had achieved good glucose control and an earlier study conducted in central Uganda that found only (26.48%) had achieved good glucose control [27]. Among the studies in Uganda that have examined glucose control and associated factors, no study has examined in a substantial way, social support from family as a possible contributing factor to achievement of optimal glycemic control.

The study reveals a significant association of perceived social support with good glucose control (p-value 0.036). These results seem to provide evidence that when there is no perceived social support from family, it is more likely to have poor glycemic control in patients with diabetes and the effect on glycemic control is more obvious than if there is strong or weak perceived social support. This finding concurs with that of a Nigeria study by Olagbemide OJ *et al*., that found a direct relationship between strong family support and glycemic control [18]. In addition, there were other important variables that were significantly associated with good glucose control at the level of bivariate analysis but lost significance at the multivariable analysis level. One of them was the inability to access a functioning glucometer to measure glucose levels as and when needed, which was noted in (25.9%) of participants. The results showed a significant association of access to a functioning glucometer with good glucose control (p-value 0.013) in this study. The proportions of those with good glucose control among those who could not access a functional glucometer was significantly lower than proportions of those with good glucose control among those who could access a functional glucometer. The possibility of measuring one´s glucose levels is a motivating factor for self-monitoring of glycemia status and taking the necessary actions depending on the result. Other studies in similar health care settings have also found that lack of self-monitoring of blood glucose and glucometer non-use are important determinants of poor glucose control [28,29]. In Uganda, inaccessibility or absence of a functional glucometer is a common finding that creates a significant challenge for achievement of optimal glucose control both at home and at the health facilities, where availability of a functioning glucometer is not consistent.

Another variable that was associated with good glucose control at bivariate analysis, was having other family members who had received some form of DSME. DSME is known to have a positive effect on DSM and glucose control for the individual [30]. This effect is more likely to be sustained when other family members acquire knowledge of facts about the disease and the skills to perform self-management tasks. They are then more likely to be of meaningful and practical help to the family member who has diabetes [31]. Most patients in this study had gotten information about diabetes and its management however; these results show that having other members of the family informed about diabetes and its management was significantly associated with good glucose control (p-value 0.012). This means that family members are likely to be more supportive in the various ways when they have knowledge about the disease and are equipped with the skills that can enable them to help family members manage their condition. These results also reveal that the use of documents to help for reference during treatment or performing other tasks related to diet and physical exercise, was significantly associated with good glucose control (p-value 0.018). Having insufficient or no information on how to appropriately manage oneself when living with the diabetes, can be a barrier to control. This also true when family members lack knowledge of diabetes to be able to influence patient´s self-management [8]. This study also shows that the presence of other chronic diseases in the same individual seemed to increase the burden of care as is shown by the observed statistical association with poor glucose control in this study (p-value <0.001). This finding is not unique to this study. In a study conducted in Burkina Faso, presence of abdominal Obesity was identified as one of the factors independently associated with prolonged poor control of Type II diabetes [19]. An Ethiopian study found that participants with comorbidities were 2.56 times more likely to have poor glucose control [28]. Also, a study conducted in the DRC found that patients with commodities are almost 3 times more likely to be uncontrolled [32]. A study by Tol *et al*., found that being married was associated with perception of support and borderline blood glucose control among Type 2 diabetic patients [20]. This study concurs with this finding in that it found a significant difference in perceived support from family in having a partner (married or cohabiting) compared to not having one [PR= 0.92 95% CI (0.87-0.98), p-value 0.008] although on adjustment for confounding, being married/cohabiting was not independently associated with perception of social support from family.

The results of multivariable analysis reveal that receiving no financial contribution from family members towards the cost of diabetic care requirements remained significantly associated with reporting perception of social support (p-value 0.043). This paradox finding could be due to the fact that majority of participants in this study reported minimal or no financial contribution from their family members. The minimal or no contribution was probably because of the limited capacity to render financial support. The high perception of support could be attributed to presence of other forms of support apart from financial support and the awareness of willingness of family members to support with whatever was available in the circumstances of limited resources. Patients living with diabetes in this kind of settings, receive support from their family members in different ways. In a qualitative study conducted in a similar context in South Africa, the participants mentioned that their family members had been supportive as regards collection of medication, food preparation, when they had sexual problems and physical activity, though some of the females participants reported that the opposite was true regarding support from their husbands [7]. Cohesion among family members in support of the patient´s care needs was significantly associated with perception of social support from family in this study. The research findings from two studies; one by Baig AA, Benitez A, Quinn MT and Burnet DL, and another by Bennich *et al*., provide evidence of impact of collaborative and supportive interactions of family members on patient´s perception of social support from family, self-efficacy, diabetes self-care [6,33]. This study found that being female was independently associated with perception of support from family (p-value 0.044). This could be explained by the females being more likely to express need for support and to get supported. This study also showed a higher proportion of females in the sample which could be related to a better health seeking behaviour than their male counter parts. It is also probable that the result is due to the imbalanced ratio of women to men in the study sample. The picture could be different with a greater proportion of male participants in the sample.

**Study limitations:** this study has contributed to a preliminary understanding of the association of social support from family members with glycemic control. However, there are some limitations; the PSS-fa questionnaire was initially developed to be self-administered but in the present study, the questions were interviewer administered which could have introduced some bias. In addition, the scale had not undergone a validation process among patients living with diabetes in Uganda despite having been used in other similar African settings with good results as far as psychometric properties are concerned. Though the study was conducted at three referral hospitals which are the highest-level health facilities in the region making the findings more likely to represent the situation in the region, generalizability could be limited due to the fact that; there are patients who do not receive care at the three hospitals; there are patients who do not speak the languages selected for this study; and the majority of patients who visit public hospitals are of low socio-economic status and therefore might not represent other patient groups. The possibility of recall bias could not be ruled out because the study involved recall of past events and circumstances. The research design of this study meant that a causal relationship could not be determined. The potential for reverse causality was still a possibility. Only outpatients were involved in this study. Admitted patients would have provided more information on the relationship being examined in the present study.

## Conclusion

Perceived social support from family was significantly associated with glycemic control. The study also found that; financial contribution from family members, cohesion among family members in support of the individual´s care and female gender were independently associated with perception of social support from family. **Recommendations:** family involvement community programs should be designed that target family members of people living with diabetes, for diabetes education. These programs could create expert patient groups to help patients cope with the stressful demands of the disease and encourage cohesion and active participation among family members in domestic activities, mobilization of financial resources in support of self-care activities that enable affected members achieve optimal glycemic control. Provision of consistent access to a functional glucometer as a necessary requirement for every diabetic at home or at nearby health facilities that can be easily accessed. This can be one effective strategy to motivate diabetic patients and their families to engage in self-monitoring of blood glucose and subsequent related actions to avoid costly disease complications and hospitalization among the affected members. A qualitative study will enrich these study´s findings by providing more detailed reasons behind perceived support among diabetic patients accessing the health care system in the eastern region of Uganda. In addition, a study to validate the PSS-fa scale, and one to examine the relationship with diabetes self-management should be done.

### What is known about this topic

- *Social support from family impacts positively on glycemic control of affected family members;*
- *Lack of social support and negative behaviour/actions from family members impacts negatively on glycemic control of affected family members*.

### What this study adds

- *Patients who feel supported by family members in managing their diabetes, are more likely to achieve good control;*
- *In resource limited settings, financial contribution and/or cohesive action from family members to provide support for management needs is an important determinant of perceived support among patients with low or no income;*
- *In addition to material and other support from family members, there is need for patients´ individual actions to have a direct effect on blood glucose levels*.
